# Reverse Split Hand as a Neurophysiological Hallmark of Spinal Muscular Atrophy

**DOI:** 10.3390/jcm13226881

**Published:** 2024-11-15

**Authors:** Veria Vacchiano, Francesca Morabito, Luigi Bonan, Luca Teodorani, Claudia Faini, Giovanni Rizzo, Rocco Liguori

**Affiliations:** 1 UOC Clinica Neurologica, IRCCS Istituto delle Scienze Neurologiche di Bologna, 40139 Bologna, Italy; mariafrancesca.morabito@ausl.bologna.it (F.M.); rocco.liguori@unibo.it (R.L.); 2 Centro Clinico NeMO, IRCCS Istituto delle Scienze Neurologiche di Bologna, 40139 Bologna, Italy; 3 Dipartimento di Scienze Biomediche e Neuromotorie, Università di Bologna, 40139 Bologna, Italy; luigi.bonan@studio.unibo.it (L.B.); luca.teodorani3@studio.unibo.it (L.T.); claudia.faini@studio.unibo.it (C.F.)

**Keywords:** MScanFit MUNE, spinal muscular atrophy, reverse split hand, amyotrophic lateral sclerosis

## Abstract

**Objective:** Motor unit number estimation (MUNE) methods are crucial for estimating lower motor neuron loss in motor neuron diseases. The MScanFit MUNE (MScanFit) is a novel method that estimates MUNE values from compound motor action potential (CMAP) scans, demonstrating high sensitivity and reproducibility in detecting motor unit loss in amyotrophic lateral sclerosis (ALS) and spinal muscular atrophy (SMA). In this study, we aimed to characterize the pattern of motor unit loss in the hand intrinsic muscles of SMA patients compared to ALS patients and healthy controls (HC) using MScanFit MUNE. **Methods:** Patients diagnosed with ALS, adult SMA patients, and HC were prospectively enrolled. MScanFit examinations were performed on the abductor pollicis brevis (APB) and abductor digiti minimi (ADM) muscles. To focus on the different patterns of motor neuron degeneration in the intrinsic hand muscles, the ratio of CMAP amplitude of APB to ADM (CMAP ratio) and the ratio of MUNE values of APB to those of the ADM muscle (MUNE ratio) were calculated. **Results:** The study included 46 ALS patients, 16 SMA patients, and 23 HC. MScanFit MUNE revealed distinct patterns of motor unit degeneration in SMA patients, notably more severe in the ADM than in the APB muscle, indicating a “reverse” split-hand phenomenon. Both CMAP and MUNE ratios demonstrated high diagnostic accuracy in distinguishing ALS from SMA, with the MUNE ratio performing better. **Conclusions:** MScanFit MUNE is a valuable tool for exploring distinct patterns of motor neuron degeneration in patients with different types of motor neuron diseases.

## 1. Introduction

Motor unit number estimation (MUNE) methods have been widely explored to assess the loss of lower motor neurons, addressing the limitations of conventional neurophysiological assessments [1,2,3,4,5,6,7]. One of the latest MUNE methods, the MScanFit MUNE (MScanFit) [7,8,9], estimates MUNE values from compound motor action potential (CMAP) scans by considering the probabilistic nature of motor unit firing and accounting for the variability of all motor units contributing to the maximal CMAP. It represents a stimulus-response relationship in which increasing the stimulus intensity gradually activates all motor units innervating the muscle, thus eliminating sampling bias. MUNE analyses are performed using the specialized QTRACW© software version 21/01/2019 (Institute of Neurology, University College London, UK, distributed by Digitimer Ltd.). This method has demonstrated high sensitivity and reproducibility in detecting motor unit loss in motor neuron diseases, such as amyotrophic lateral sclerosis (ALS) [10,11] and spinal muscular atrophy (SMA) [12,13,14]. Furthermore, it is semi-automated and quick to perform. 

ALS and SMA are both motor neuron diseases, but they have distinct underlying pathological mechanisms. Specifically, ALS is an adult-onset, usually sporadic, neurodegenerative disease characterized by the progressive loss of both upper and lower motor neurons [15]. Although various factors, such as genetic influences, disruptions in protein homeostasis, abnormalities in RNA metabolism, mitochondrial dysfunction, glutamate-induced excitotoxicity, and impaired intracellular transport in neurons, may contribute to the pathogenesis of ALS, the exact underlying mechanism remains unclear [15].

SMA is an autosomal recessive genetic disorder, marked by the degeneration of lower motor neurons due to a deficiency of the survival motor neuron (SMN) protein. The clinical presentation of SMA is highly variable, with different levels of severity. It is classified into four subtypes based on the age of symptom onset (ranging from infancy to adulthood) and the motor milestones achieved [16].

The split-hand phenomenon reflects the disproportionate atrophy and weakness of the muscles on the lateral side of the hand compared to the hypothenar eminence muscles. Understanding this phenomenon is crucial for diagnosing and monitoring motor neuron diseases. While there is extensive literature on this phenomenon in ALS [17,18,19], using various neurophysiological methods [20,21], knowledge remains limited for other motor neuron diseases, including SMA. The mechanisms underlying this dissociated pattern of muscle dysfunction are still unclear. In ALS, axonal excitability has been found to be higher in the median motor axons innervating the APB compared to the ulnar motor axons innervating the ADM muscle [18]. Additionally, cortical hyperexcitability is more pronounced when recorded over the thenar muscles than the ADM muscles, suggesting that an imbalance in cortical excitability among hand muscles may contribute to the split-hand phenomenon [19]. Thus, both cortical and peripheral hyperexcitability may play a role in the development of this phenomenon.

So far, only one study had focused on the split hand in both ALS and SMA patients using the motor unit number index (MUNIX) [22]. In our study, we aimed to characterize the pattern of motor unit loss in SMA patients compared to those with ALS, the most prevalent motor neuron disease, as well as healthy controls, using MScanFit MUNE.

## 2. Materials and Methods

### 2.1. Subjects and Clinical Variables 

Patients diagnosed with ALS according to the Revised El Escorial criteria [23], adult patients with genetically confirmed SMA, and healthy controls were prospectively enrolled between January 2021 and May 2024.

Clinical data from patients were collected at the time of neurophysiological investigations. For ALS patients, the following variables were recorded: age, sex, time, and type of onset (bulbar vs. spinal), clinical phenotype [24], revised amyotrophic lateral sclerosis functional rating (ALSFRS-R) scale, and the forced vital capacity (FVC). We also calculated the disease progression rate (DPR), defined as 48 minus the ALSFRS-R score at the time of the MScanFit examination, divided by the disease duration.

We categorized the different types of SMA according to the age of symptom onset and the motor milestones achieved, in line with the standard classification system [16]. The duration of each disease was calculated as the time elapsed from the first reported symptom to the MScanFit assessment, measured in months.

Exclusion criteria for patients and healthy controls were documented median and ulnar nerve lesions, polyneuropathy or diseases that could induce polyneuropathy, presence of pacemaker, and dementia.

### 2.2. MScanFit MUNE

MScanFit assessments were performed on the APB and ADM muscles of all participants. In healthy subjects, the dominant side was evaluated, whereas in ALS and SMA patients, the less affected side was examined.

The set-up for CMAP scan recordings included a DS5 bipolar stimulator (Digitimer, Welwyn Garden City, UK), a D440-2 amplifier (Digitimer, Welwyn Garden City, UK), and a 50 Hz HumBug noise eliminator (Digitimer, Welwyn Garden City, UK) controlled by QtracW software (Institute of Neurology, University College London, UK) [25]. The filter settings were 3 Hz–10 kHz. The manufacturer of recording and stimulating electrodes was Ambu BlueSensor (Bayan Lepas, Penang, Ambu, Malaysia).

For the median nerve, the active recording electrode was positioned over the APB muscle and the reference electrode on the first digit’s metacarpophalangeal joint. Stimulation was applied at the wrist, between the flexor carpi radialis and palmaris longus tendons. For the ulnar nerve, the active recording electrode was placed on the ADM muscle, with the reference electrode on the fifth digit’s metacarpophalangeal joint and stimulation at the wrist between the flexor carpi ulnaris and palmaris longus tendons.

Recordings were conducted using the MScan-R2 protocol [25]. Subjects were instructed to remain silent and as relaxed as possible during the examination. The stimulus duration was set to 0.2 ms. The stimulus intensity was increased until supramaximal stimulation, at which point the CMAP scan was initiated. This sequence ran automatically with 20 pre-scan stimuli at supramaximal intensity. The intensity was then gradually reduced by 0.2% steps every 0.6 s until the motor response could no longer be recorded. Finally, 20 low-intensity post-scan stimuli were applied before the recording was terminated.

Using the offline MScanFit component of the QtracP analysis program, a model was fitted to the recorded stimulus–response curve (CMAP scan) to estimate the motor unit number, as well as the distribution of motor unit sizes and thresholds [7].

Baseline-to-peak amplitude was measured for supramaximal CMAP values.

Motor unit number estimates were then calculated offline using the MScanFit facility in the QtracP software version 21/01/2019 (updated version MScanFit-2). In brief, the process begins with generating a preliminary model based on the slope and variance of the scan points; the model is then refined by adjusting all the parameters to enhance the fit between the original scan and the scans generated by the model. The following parameters were derived from the automatic analysis [26].

The parameters reflecting the extent of the degeneration are as follows:

- MUNE: the estimated number of functional motor units.
- N50: the estimated number of larger units making up 50–100% of the amplitude of the CMAP.

The parameters reflecting the phenomenon of collateral reinnervation are as follows:

- HalfAmpAmp (A50): the size of the motor units at the 50% mark of the cumulative amplitude expressed in % (it is an amplitude measure designed to be less sensitive to the size limit than mean or median amplitude).
- Largest single motor unit potential (LSMUP): the amplitude of the largest unit, expressed as a percentage of CMAP amplitude.

The protocol was approved by the local Medical Ethical Committee (project identification code 23074, approved on 31 May 2023). Informed consent was obtained from all participants.

### 2.3. Statistical Analysis

Statistical analysis was performed using the software QtracP (©Institute of Neurology, University College London) and IBM SPSS Statistics version 27 (IBM, Armonk, NY, USA). For continuous variables, the Mann–Whitney U test or the Kruskal–Wallis test was used to perform group comparisons. The chi-square test was adopted for categorical variables. Since ALS patients were significantly older compared to both SMA patients and healthy subjects, general linear models were employed, including disease type as the independent variable, neurophysiological parameters as the dependent variables, and age as a covariate to adjust for its potential confounding effect. In order to focus on the different patterns of motor neuron degeneration of the intrinsic hand muscles, we calculated the ratio of the maximal CMAP amplitude of APB to that of ADM (CMAP ratio) and the ratio of MUNE values of APB to those of ADM muscles (MUNE ratio). Receiving operator characteristic (ROC) curve analysis was conducted to determine the area under the curve (AUC) and cut-off values of both CMAP and MUNE ratios for distinguishing between ALS and SMA patients.

Sensitivity, specificity, and the area under curve (AUC) were calculated. *p* values < 0.05 were considered as statistically significant.

## 3. Results

### 3.1. Clinical Features of the Study Population 

A total of 46 ALS patients, 16 SMA patients, and 23 healthy controls were enrolled in the study. There were no statistical differences in age (*p* = 0.16) between SMA patients and healthy controls. ALS patients were significantly older than both SMA patients (*p* < 0.001) and controls (*p* < 0.001). The distribution of sex was not significantly different between the three groups (*p* = 0.3). ALS patients were classified according to the revised El Escorial criteria [23] as clinically possible (*n* = 7), clinically probable, laboratory-supported (*n* = 13), clinically probable (*n* = 17), and clinically definite (*n* = 9) ALS. SMA patients were categorized as type 2 (*n* = 3) and type 3 (*n* = 13). Table 1 summarizes the demographic and clinical features of the study population.

### 3.2. MScanFit MUNE Parameters Across Population Study

APB and ADM muscles were assessed using MScanFit MUNE in all subjects. Group comparisons of neurophysiological parameters derived from MScanFit for ALS patients, SMA patients, and healthy subjects using the Kruskal–Wallis test are shown in Table 2.

A general linear model adjusted for age showed that CMAP values of the APB muscle were significantly lower in ALS and SMA patients compared to controls (*p* < 0.01), while values were quite similar between ALS and SMA patients (*p* = 0.701). MUNE and N50 values from the APB muscle were higher in healthy controls compared to ALS and SMA patients (*p* < 0.001). Furthermore, these values were lower in SMA patients compared to ALS patients (*p* = 0.03 and *p* = 0.045, respectively). The LSMUP and A50 values were significantly lower in healthy subjects compared to both disease groups (*p* < 0.02) and were not significantly different between ALS and SMA patients (*p* = 0.707 and *p* = 0.295). 

Focusing on the ADM muscle, a general linear model showed that CMAP values were significantly lower in SMA and ALS patients compared to controls (*p* < 0.001) and lower in SMA patients than in ALS patients (*p* = 0.025). Similarly, MUNE and N50 values were lower in both disease groups compared to controls (*p* < 0.001) and were lower in SMA patients than in ALS patients (*p* < 0.001). Likewise, LSMUP and A50 were higher in both disease groups than controls (*p* < 0.05) and in SMA patients than ALS patients (*p* < 0.001). 

### 3.3. Pattern of Motor Unit Loss in Intrinsic Hand Muscles

To focus on the different patterns of motor neuron degeneration of the intrinsic hand muscles, we calculated the ratio of the maximal CMAP amplitude of the APB to that of the ADM (CMAP ratio) and the ratio of MUNE values of the APB to those of the ADM muscles (MUNE ratio). Applying the previously suggested cut-off value of 0.6 for the CMAP ratio [17], abnormal values (i.e., <0.6) were observed in 21/46 ALS patients (45.6%), 2/23 (8.7%) of healthy controls, and none of the SMA patients (*p* < 0.001).

**Figure 1:**
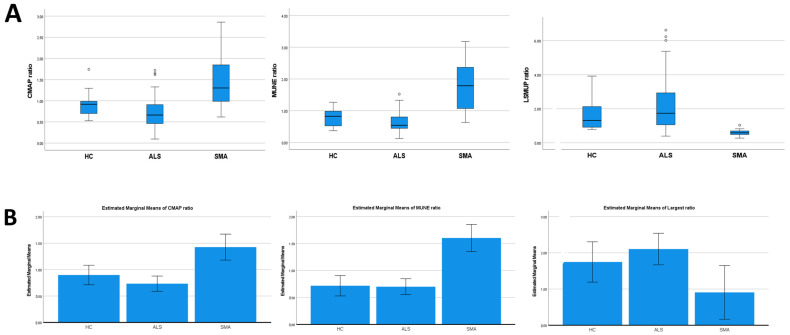
(**A**): Group comparisons were implemented using the Kruskal–Wallis test. The CMAP ratio was significantly higher in SMA patients than in controls (*p* = 0.017) and ALS patients (*p* < 0.001), and it was lower in ALS patients than in controls (*p* = 0.014). The MUNE ratio was higher in SMA patients than in both ALS patients and controls (*p* < 0.001), and it was lower in ALS patients than in controls (*p* = 0.047). (**B**): General linear models adjusted for age. The CMAP ratio was significantly higher in SMA patients than in ALS patients and healthy controls (*p* < 0.001), but it was not significantly different between ALS patients and controls (*p* = 0.199). The MUNE ratio was significantly higher in SMA patients than in ALS patients and controls (*p* < 0.001), while it was not different between ALS patients and controls (*p* = 0.89). The LSMUP ratio was significantly lower in SMA patients than in ALS patients (*p* = 0.014) and showed a trend toward significance compared to controls (*p* = 0.05) (**B**).

Group comparisons using the Kruskal–Wallis test showed that the CMAP ratio was significantly higher in SMA patients than in controls (*p* = 0.017) and ALS patients (*p* < 0.001). Furthermore, these values were lower in ALS patients than in controls (*p* = 0.014). Similarly, the MUNE ratio was higher in SMA patients than in both ALS patients and controls (*p* < 0.001) and lower in ALS patients than in controls (*p* = 0.047) (Figure 1A).

A general linear model adjusted for age confirmed that the CMAP ratio was significantly higher in SMA patients than in ALS patients and healthy controls (*p* < 0.001), but it did not confirm the difference between ALS patients and controls (*p* = 0.199). Similarly, the MUNE ratio was significantly higher in SMA patients than in ALS patients and controls (*p* < 0.001), while it was not different between ALS patients and controls (*p* = 0.89) (Figure 1B).

To focus on the different patterns of reinnervation, we calculated the ratio of the LSMUP of the APB to that of the ADM muscle (LSMUP ratio). We observed that the values were significantly lower in SMA patients compared to both ALS patients and healthy controls (*p* < 0.001), but they were comparable between SMA and ALS patients (*p* = 0.314), Figure 1A. After adjusting for age, the LSMUP ratio was significantly lower in SMA patients than in ALS patients (*p* = 0.014) and showed a trend toward significance compared to controls (*p* = 0.05) (Figure 1B).

**Figure 2:**
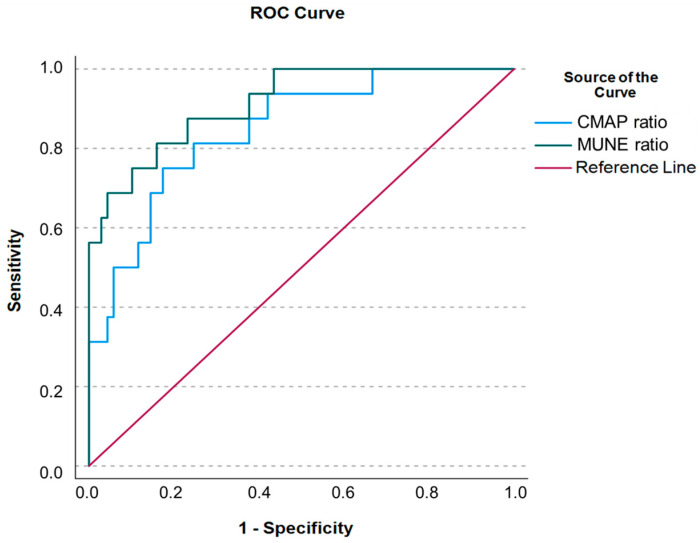
ROC curve analysis to determine the AUC and cut-off values for both CMAP and MUNE ratios for distinguishing between ALS and SMA patients. CMAP ratio: AUC = 0.871 (95% CI: 0.779–0.963), sensitivity = 93.8%, specificity = 69.6%, and cut-off = 0.79 (*p* < 0.001). MUNE ratio: AUC = 0.931 (95% CI: 0.867–0.995), sensitivity = 93.8%, specificity = 71.7%, and cut-off = 0.69 (*p* < 0.001).

ROC curve analysis was conducted to determine the area under the curve (AUC) and cut-off values for both CMAP and MUNE ratios for distinguishing between ALS and SMA patients. We found that the AUC for the CMAP ratio was 0.871 (95% CI: 0.779–0.963), with a sensitivity of 93.8%, a specificity of 69.6%, and a cut-off of 0.79 (*p* < 0.001). The MUNE ratio showed a better performance, with an AUC of 0.931 (95% CI: 0.867–0.995), a sensitivity of 93.8%, a specificity of 71.7%, and a cut-off of 0.69 (*p* < 0.001) (Figure 2). 

**Figure 3:**
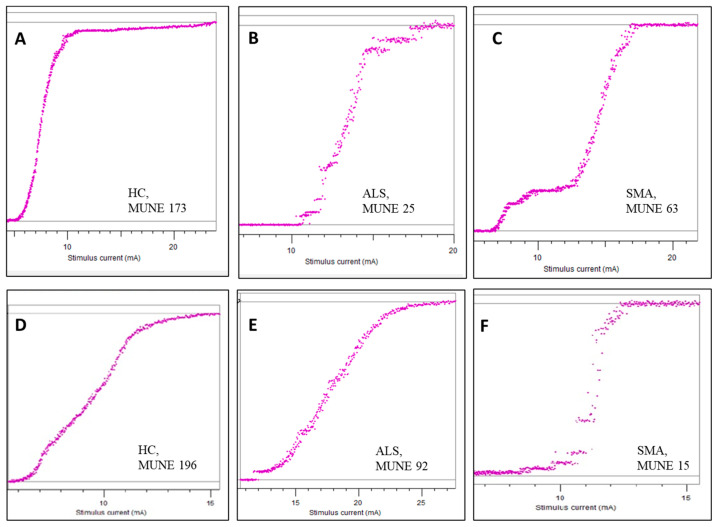
MUNE values of the APB muscle in a healthy control (**A**), an ALS patient (**B**), and a SMA subject (**C**). MUNE values of the ADM muscle in a healthy control (**D**), an ALS patient (**E**), and a SMA subject (**F**). On the horizontal axis, the stimulus current (mA) is displayed; on the vertical axis, the CMAP amplitude (mV) is displayed. Note the reverse pattern in the SMA patient, with MUNE 63 in the APB vs. MUNE 15 in the ADM muscle.

An illustrative example of MscanFit applied to the APB and ADM muscles in an ALS patient, an SMA subject, and a healthy control is shown in Figure 3.

### 3.4. Clinical-Neurophysiological Correlations Across Motor Neuron Diseases

In the ALS cohort, neurophysiological parameters including CMAP, MUNE values, and reinnervation parameters from the APB and ADM muscles, as well as the CMAP and MUNE ratios, did not differ between types of onset, clinical phenotypes, or diagnostic categories21 (*p* > 0.05). Age at examination correlated only with MUNE (Rho = 0.31, *p* = 0.038), LSMUP (Rho = 0.29, *p* = 0.048), and A50 (Rho = 0.37, *p* = 0.011) of the APB muscle, and with the MUNE ratio (Rho = −0.3, *p* = 0.041). Disease duration, DPR, ALSFRS-R, and its motor subdomains (items 4–9) did not correlate with any neurophysiological parameters or with MUNE and CMAP ratios. FVC was correlated with CMAP and MUNE values of the ADM muscles (Rho = 0.45, *p* = 0.003 and Rho = 0.31, *p* = 0.047, respectively).

In the SMA cohort, age and disease duration did not correlate with neurophysiological parameters. Group comparisons using the Mann–Whitney test showed that SMA type 2 patients had lower CMAP and MUNE values (*p* = 0.014 and *p* = 0.025, respectively) and higher LSMUP and A50 values of the APB muscle (*p* = 0.039 and *p* = 0.025, respectively) compared to SMA type 3 patients. Furthermore, CMAP values from the ADM muscle were lower in SMA type 2 patients than in type 3 patients (*p* = 0.025), while MUNE values did not differ significantly (*p* = 0.11). Conversely, A50 and LSMUP values were higher in SMA type 2 patients than in type 3 patients (*p* = 0.025 and *p* = 0.082, respectively). CMAP and MUNE ratios did not differ among SMA types (*p* > 0.05).

## 4. Discussion

In this study, we used the MScanFit MUNE method to characterize the pattern of motor unit loss in the intrinsic muscles of the hand in two different motor neuron diseases (ALS and SMA), compared to healthy controls.

We observed a distinct pattern of motor unit degeneration in the hands of SMA patients, characterized by more severe involvement of the ADM compared to the APB muscle, which was diametrically different from that observed in ALS patients. Indeed, apart from the CMAP value, parameters derived from MScanFit reflecting both neurodegeneration (MUNE and N50) and reinnervation (A50 and LSMUP) suggested a more significant impairment of the ADM in SMA compared to ALS patients. This “reverse” split-hand phenomenon was also evident using both CMAP and MUNE ratios, with significantly lower values observed in ALS patients compared to those with SMA.

Furthermore, both CMAP and MUNE ratios exhibited very good diagnostic accuracy in distinguishing between ALS and SMA patients, with the MUNE ratio showing slightly better performance. Interestingly, the reverse split-hand phenomenon in SMA patients has been described in other studies, which used the MUNIX on intrinsic hand muscles of patients with SMA [22,27,28]. Similarly to our study, the authors did not find any correlation with disease duration and investigated the less affected side [22], suggesting that this pattern is present even in the early phase of the disease, supporting its use as an “ideal diagnostic tool”. Furthermore, the reverse split hand was also reported in pediatric SMA patients [28], reinforcing its early manifestation.

We confirmed these observations by using the MScanFit MUNE, which overcomes the limitations of other MUNE methods, including MUNIX, by obtaining a representative sample of motor units not based on voluntary effort [7]. Additionally, MScanFit MUNE provided interesting information not only on the estimation of the preserved functional motor units but also on the mechanism of compensatory nerve sprouting, which was more evident in the ADM muscle than in the APB muscle in SMA patients compared to both healthy controls and ALS patients, in contrast with previous MUNIX findings [28].

Notably, the reverse split hand has been reported in another non-ALS motor neuron disease, the brachial monomelic amyotrophy [29,30], possibly as a result of a predominant C8 myotomal involvement due to cord compromise at this level, with a relative spare of T1 (primarily innervating the APB muscle). To the best of our knowledge, no other studies have demonstrated a pattern consistent with the reverse split hand in other non-ALS motor neuron diseases [30].

In our study, MUNE and CMAP ratios were significantly lower in ALS patients compared to healthy controls, confirming the presence of split hands in ALS. However, this pattern was not confirmed after adjusting for age. One possible reason is that we examined the less affected side of ALS patients in the early stage of the disease. Additionally, another explanation could be the physiological phenomenon of preferential atrophy of the lateral aspect of the hand with aging, resulting in a “physiological split-hand” due to the aging process [31,32]. Indeed, previous studies showed that some neurophysiological ratios proposed to assess the split-hand phenomenon, such as the APB to ADM CMAP ratio, exhibited negative correlations with age [31,32,33]. Consistently, we observed in our entire cohort a negative correlation between the age of subjects and both CMAP and MUNE ratios (Rho = −0.43, *p* < 0.001 and Rho = −0.52, *p* < 0.001, respectively), endorsing previous findings. This may reflect the specific susceptibility of certain muscles during the normal aging process of the hand, possibly due to cumulative stress on motor axons from repetitive precision or pincer grip use [29]. This aging pattern may partially overlap with that observed in the upper limbs of ALS patients, potentially leading to an overestimation of the extent of neurodegeneration in elderly patients with ALS. However, our relatively small cohort of healthy controls limits us from drawing definitive conclusions.

While we confirmed the correlation between MScanFit MUNE neurophysiological parameters and disease severity in SMA patients, we did not find convincing correlations with clinical variables in ALS patients, particularly with the ALSFRS-R scale. On this issue, although several studies have reported the correlation between MUNE values and the ALSFRS-R scale [8,11], others have not confirmed it [21,34]. This discrepancy may result from the nature of the scale, which reflects overall functional status rather than the specific performance of the neurophysiologically examined muscles. Furthermore, CMAP and MUNE ratios were comparable between patients with the bulbar or spinal onset and across different clinical phenotypes, in line with previous findings [30].

Our study has some limitations. First, we enrolled a relatively small number of SMA patients, which might reduce the robustness of our results; however, this is expected given the rarity of the disease.

Furthermore, we did not conduct longitudinal observations, which would have enhanced the effectiveness of MScanFit MUNE in monitoring motor neuron diseases.

Overall, we demonstrated that using MScanFit MUNE to assess the hand is an objective, non-invasive tool for early differential diagnosis. Understanding the preferential involvement of the medial hand in SMA patients could aid in selecting the best muscle target for monitoring disease progression and treatment response, especially in severe cases where certain neurophysiological indices, like ADM CMAP, are difficult to obtain. In this regard, while ADM CMAP may be sensitive in detecting disease onset in presymptomatic newborns [35,36], recent studies suggest that APB CMAP is a better biomarker for assessing therapeutic response [37,38]. Our results confirm that the APB muscle might be the optimal target for monitoring disease progression and therapeutic response in SMA patients, as it is less susceptible to motor neuron degeneration.

In future studies, it will be important to ascertain whether this “reversed split hand” phenomenon is unique to SMA or if it also occurs in other non-ALS motor neuron disorders, as well as to determine the reason for this peculiar focal muscle susceptibility.

## Figures and Tables

**Table 1:** Demographic and clinical features of the study population.

ALS Patients	N (tot. 46)	%
*Sex*	27 (M)	58.7
*Type of onset*		
Bulbar	14	30.4
Spinal	32	69.6
*ALS phenotype*		
Classic	33	71.7
Bulbar	8	17.4
PLMN	3	6.5
PUMN	2	4.3
		Median (IQR)
*Age at enrolment (y)*		69.5 (58–73.3)
*Age at onset (y)*		68 (57.8–73)
*Disease duration (months)*		10.5 (6–19.5)
*ALSFRS-R score*		42 (39–44.5)
*ALSFRS-R motor subscore*		20 (17–22)
*FVC*		89.5 (74.8–107.8)
**Healthy controls**	**N (tot. 23)**	**%**
*Sex*	9 (M)	39.1
		Median (IQR)
*Age at enrolment (y)*		53 (24–59)
**SMA patients**	**N (tot. 16)**	**%**
*Sex*	8 (M)	50
*SMA type*	13 (type 3)	81.3
	3 (type 2)	18.8
		Median (IQR)
*Age at enrolment (y)*		32.5 (19.3–47.3)
*Disease duration (months)*		357 (208–448)

ALS, amyotrophic lateral sclerosis; ALSFRS-R, revised amyotrophic lateral sclerosis functional rating; FVC, forced vital capacity; IQR, interquartile range; M, males; PLMN, predominant lower motor neuron; PUMN, predominant upper motor neuron; SMA, spinal muscular atrophy; y, years.

**Table 2:** Group comparisons of neurophysiological parameters derived from MScanFit for ALS patients, SMA patients, and healthy subjects using the Kruskal–Wallis test.

	APB			*p*-Value	ADM			*p*-Value
	ALS	SMA	HC		ALS	SMA	HC	
CMAP peak (mV)	4.1 (0.4; 2.4–6.1)	6.4 (0.9; 2.2–8.9)	9.1 (0.5; 6.9–10.7)	<0.001 * 0.106 ** <0.001 *** 0.005 #	6.2 (0.4; 4.2–9.3)	4.7 (0.9; 1.7–8.6)	9.8 (0.3; 8.6–11.1)	<0.001 * 0.11 ** <0.001 *** <0.001 #
MUNE	32.5 (4.1; 25.3–61.3)	34.5 (6.9; 20–47.5)	92 (5.7; 80–128)	<0.001 * 0.575 ** <0.001 *** <0.001 #	74.5 (5.9; 43.8–105)	22 (4.8; 11–32.8)	134 (6.8; 114–171)	<0.001 * <0.001 ** <0.001 *** <0.001 #
N50	9.1 (1.1; 4.6–15.3)	7.9 (1.9; 3.7–12.9)	25.7 (2.1; 17.9–31.6)	<0.001 * 0.588 ** <0.001 *** <0.001#	16.2 (1.9; 10–27.6)	4.5 (1.1; 2.2–8.4)	42.9 (2.9; 29.4–49.2)	<0.001 * <0.001 ** <0.001 *** <0.001 #
A50 (%)	3.7 (0.8; 2–6.6)	3.8 (1.1; 2.7–7.6)	1.2 (0.1; 0.9–1.5)	<0.001 * 0.507 ** <0.001 *** <0.001 #	1.8 (0.5; 1.1–2.9)	5.9 (1.5; 3.9–13.1)	0.8 (0.1; 0.7–1)	<0.001 * <0.001 ** <0.001 *** <0.001 #
LSMUP (%)	10.1 (1.7; 6.3–20.2)	9.8 (2.2; 7.8–17.9)	3.7 (0.3; 3–5.2)	<0.001 * 0.69 ** <0.001 *** <0.001 #	6.5 (1; 3.6–11.2)	18.4 (3.5; 11.6–30.1)	2.8 (0.2; 2–3.4)	<0.001 * <0.001 ** <0.001 *** <0.001 #

Values are expressed as median (mean standard error; interquartile range). Key: ADM, abductor digiti minimi; ALS, amyotrophic lateral sclerosis; APB, abductor pollicis brevis; A50, the size of the motor units at the 50% mark of the cumulative amplitude expressed in %; CMAP, compound motor action potential; HC, healthy controls; MUNE, motor unit number estimation; LSMUP, amplitude of the largest unit, expressed as a percentage of CMAP amplitude; mV, millivolt; N50, estimated number of larger units making up 50–100% of the amplitude of the CMAP; SMA, spinal muscular atrophy. * *p*-values refer to the comparison between ALS, SMA patients, and healthy controls (Kruskal–Wallis test). ** *p*-values refer to pairwise comparisons between ALS and SMA patients (Kruskal–Wallis test). *** *p*-values refer to pairwise comparisons between ALS and healthy controls (Kruskal–Wallis test). # *p*-values refer to pairwise comparisons between SMA and healthy controls (Kruskal–Wallis test).

## Data Availability

The data that the findings of this study are available from the corresponding author upon reasonable request.
